# Effects of a Finger Tapping Fatiguing Task on M1-Intracortical Inhibition and Central Drive to the Muscle

**DOI:** 10.1038/s41598-018-27691-9

**Published:** 2018-06-19

**Authors:** Antonio Madrid, Elena Madinabeitia-Mancebo, Javier Cudeiro, Pablo Arias

**Affiliations:** 1 Universidade da Coruña, NEUROcom (Neuroscience and Motor Control Group) and Biomedical Institute of A Coruña (INIBIC), Department of Biomedical Sciences, Medicine and Physiotherapy-INEF Galicia, A Coruña, Spain; 2 Centro de Estimulación Cerebral de Galicia, A Coruña, Spain

## Abstract

The central drive to the muscle reduces when muscle force wanes during sustained MVC, and this is generally considered the neurophysiological footprint of central fatigue. The question is if force loss and the failure of central drive to the muscle are responsible mechanisms of fatigue induced by un-resisted repetitive movements. In various experimental blocks, we validated a 3D-printed hand-fixation system permitting the execution of finger-tapping and maximal voluntary contractions (MVC). Subsequently, we checked the suitability of the system to test the level of central drive to the muscle and developed an algorithm to test it at the MVC force plateau. Our main results show that the maximum rate of finger-tapping dropped at 30 s, while the excitability of inhibitory M1-intracortical circuits and corticospinal excitability increased (all by approximately 15%). Furthermore, values obtained immediately after finger-tapping showed that MVC force and the level of central drive to the muscle remained unchanged. Our data suggest that force and central drive to the muscle are not determinants of fatigue induced by short-lasting un-resisted repetitive finger movements, even in the presence of increased inhibition of the motor cortex. According to literature, this profile might be different in longer-lasting, more complex and/or resisted repetitive movements.

## Introduction

Muscle fatigue induced by sustained contractions may develop at the muscle but also at neural levels (i.e., central fatigue)^1^. The twitch-interpolation technique evaluates central fatigue by assessing the level of central drive to the muscle (also called voluntary activation, VA). It involves a first percutaneous electric stimulation (PNS) of a nerve (or muscle motor point) during a maximal voluntary contraction (MVC), which normally increases the ongoing force-torque developed by the muscle (interpolated twitch). The twitch size depends on the amount of axons that the subject was not voluntarily activating and on the firing rate of those others discharging. When this magnitude is expressed as a fraction of the force-torque subsequently induced at rest by a second PNS, the VA is estimated^2^. The VA reduces when muscle force wanes during sustained MVC^1^, and this is generally considered the footprint of central fatigue. Remarkably, fatigue is task-dependent^3^, and in the case isometric activities the central origin of fatigue has been thoroughly studied. However, some important activities of daily living potentially fatiguing at central level are not isometric. The question to be answered is if the failure of central drive to the muscle is a responsible mechanism of fatigue induced by different activities than those involving isometric contractions.

Rhythmic repetitive movements are also fundamental in activities of daily living and require low levels of muscle force (gait, typewriting, etc). Understanding the central underpinnings of fatigue in these movements are of paramount importance at basic and clinical levels. A model used worldwide to test repetitive movements is the finger tapping (*ft*) test. *Ft* permits the characterization of bradykinesia, hypometria and arrythmkinesia in a number of diseases^4^. Its execution at maximal rates for 10–30 secs reduces its frequency rapidly^5–8^ and increases primary motor cortex (M1)-intracortical inhibition^5,8^, which suggests the presence of central fatigue. Notwithstanding, the rate-drop during 10–30 secs of *ft* at maximal rate is larger than MVC force-drop tested right after *ft*^6,8,9^. For these reasons, we suggest that force and central drive to the muscle might not be key elements of neural adaptations of fatiguing repetitive movements. In addition to central fatigue, fatigue at peripheral level also reduces muscle performance. Peripheral expressions of fatigue include a whole variety of mechanisms which might compromise the efficiency of the neuromuscular transmission, muscle excitation or muscle contraction dynamics^10^. The muscle contractile properties, which can be tested with the velocity of muscle relaxation^10,11^, appear to be less affected during isometric than concentric muscle fatiguing contractions^12^; likely, it is a limiting factor reducing the frequency of movement during fast rate *ft*.

Our hypothesis is that failure of central drive to the muscle is not amongst the central mechanisms responsible for the inability to maintain the maximal rate during *ft*, despite the increased inhibition of the motor cortex observed at the end of this repetitive task^5,8,9^.

## Methods

To test our hypothesis, we ran several experimental blocks (EBs). Preliminarily, we designed and tested a 3D-printed hand-fixation system, enabling the execution of *ft* and MVC. We also developed an algorithm to deliver stimulation at the MVC plateau; this permitted us to minimize the duration of the MVC for testing VA, which could potentially interfere with expressions of central fatigue previously induced by *ft*. Then, we checked the suitability of the system to evaluate the VA by means of the twitch-interpolation technique. These preliminary methods and results are presented in *Supporting Information*. The protocols followed the Declaration of Helsinki recommendations and were approved by the University of A Coruña Ethics Committee. Participants signed informed consent forms.

### Participants

Eleven healthy participants completed all EBs (age range [19–47 yrs]; all participants were men and right-handed^13^). They were screened for incompatibility with brain stimulation techniques.

### Material

The 3D-printed hand-fixation system permitted the adaptation of a force sensor (P200, Biometrics Ltd, Newport, UK, NP11 7 HZ) to evaluate the force applied by the distal phalange of index finger towards flexion or abduction around the metacarpophalangeal joint while the thumb was secured in abduction^14^ (see Supporting Sketch Files and Supporting Fig. 1 for full description of hand position and fixation). A 2-axis goniometer (SG100 Biometrics Ltd) monitored flexo-extension and abduction-adduction movements around the index-finger metacarpophalangeal joint. Sensors were connected to a K800 amplifier (Biometrics Ltd) that communicated with a CED-1401 mkII (unit-1). This unit was controlled with Signal 4 software to sample signals and present event-related stimuli (light emitting diodes –LED- turning on/off); these were cues for the subject to indicate the different phases of the tasks (Supporting Video).

A Digitimer D360 amplifier acquired electromyographic activity from the superficial head of the *first dorsal interosseous* muscle (gain x200-1000, between 3–3000 Hz) and sent the signal to the CED-unit-1 (sampling at 10 KHz). This head of the *first dorsal interosseous* muscle is a specific flexor of the index finger if the thumb is fixed in abduction^14^. Our preliminary EB clearly confirmed that PNS of the *first dorsal interosseous* muscle supplying nerve (ulnar), with the thumb fixed in abduction, produced a prominent flexion of the index metacarpophalangeal joint (see description below and Supporting Fig. 1). We excluded from the study other index flexors acting also in other fingers (like the *flexor digitorum supperficialis*)^15^ since their activity was not repetitive in our task. Also, the extended wrist position during testing (Supporting Fig. 1) stretches the flexors crossing the joint; in these conditions spinal motoneurons innervating these muscles are inhibited at pre-synaptic and post-synaptic levels^16^. *Ft* at the fastest rate alternates active flexion and extension movement phases; for these reason the selection of the *first dorsal interosseous* muscle is suitable for our purposes.

The force signal from the K800 amplifier was also sent (in parallel) to another CED-1401 mkII (unit-2) controlled with Sequencer software and sampled at 100 KHz. Sequencer software ran a customized algorithm to deliver TTL pulses and trigger stimulators when the MVC force-peak was reached and the plateau just started (Supporting Figs 2–3 and Video; the Supporting Video is a demonstrative movie, separated from the experimental sessions, to display the participant’s execution and the behavioural recordings: it includes the last seconds of 30 s *ft* task, the execution of a MVC just at the end of *ft*, and the PNS to calculate the VA).

### Protocols

In a Preliminary-EB, we evaluated the suitability of our setup to test the VA by means of the twitch-interpolation technique. To do so, we recorded the index-finger force magnitude (towards flexion and abduction) produced by a triplet supramaximal (PNS) of the ulnar nerve (Supporting Fig. 1).

In the *ft* session of the main EB, we evaluated how 30 s of *ft* at the maximal possible rate modifies the level of central fatigue (tested with no gap or resting time from *ft*); this evaluation was done repeatedly in 8 sets with 3 min and 30 s inter-set interval. The *control session* of this EB was identical to the *ft session*, except that *ft* was not executed, but the MVC and central fatigue were tested with the same timing as in the *ft session*. With the *control session*, we evaluated whether the execution of MVC over time during the *ft session* might have influenced the level of central fatigue^17^.

Once the *control session* was finished, subjects executed a complementary EB including 20 brief MVCs (with 20 s inter-repetition interval), and during each MVC we tested the VA. The protocols are detailed in the following sections.

### Preliminary EB: Suitability of the setup to test the VA

While the subject was at rest and the muscle was in a fresh un-potentiated state, we applied PNS. Stimulation was a triplet (100 Hz) at supramaximal intensity; 150% of intensity needed to get the maximal compound muscle action potential (CMAP) in the *first dorsal interosseous* muscle. This was applied with a Digitimer DS7AH (0.2 ms pulse-widths), with the anode lateral to the medial epicondyle of the humerus along the post-condylar groove and the cathode approximately 2 cm distal to the anode along the direction of the nerve (this configuration was applied in all EBs). Force and electromyographic signals were recorded and the magnitude of the force twitch generated by the stimulation was analysed. Five twitches were acquired at 20 second intervals for testing the force generated by the stimulation towards index flexion and another 5 twitches towards index abduction (changing the position of the sensor but maintaining the position of the finger; see Supporting Sketch Drawings and Supporting Fig. 1). The order to test abduction or flexion was counterbalanced across participants.

### Main EB. Ft session

Subjects executed 8 sets of 30 seconds of *ft* (flexo-extension) on the force sensor; they were encouraged to execute at their maximal possible rate in all sets. In each set, subjects executed 2 brief MVCs (towards flexion) with feed-back provided. The signal for the first MVC was the lighting of a red LED; MVC stopped when the LED went out (500 ms after delivering the stimulation at MVC); the recorded variables at this time are referred as *pre* in the text. After 18 s, *ft* started. Right after 30 seconds of *ft* (*post*), the LED turned on again and subjects changed from *ft* to MVC without resting time (Supporting Video). The instructions given to the subjects were “*tap with the index finger at your maximum possible rate from the very beginning of the test until the LED turns on, and at that moment, press as fast and hard as you can for as long as the LED remains on”*.

**Figure 1:**
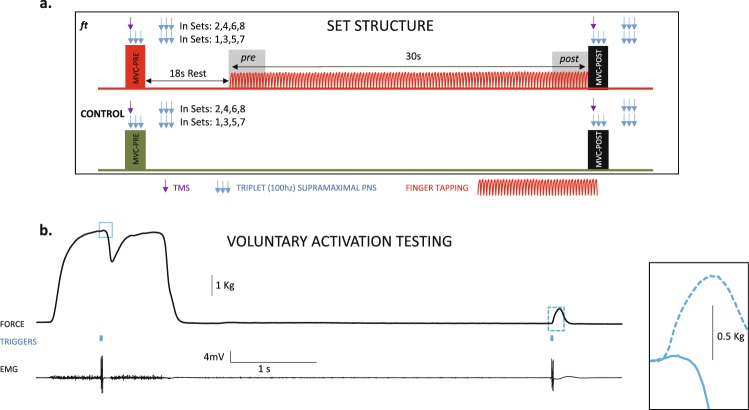
Set structure for ft and control session and VA testing. (**a**) The *ft* and *control* sessions included 8 sets. The set started with an MVC-*pre*; after 18 sec of rest, subjects executed *ft* as fast as possible for 30 sec, which continued with an MVC-*post* (in red colour). From the grey-shaded areas (4 secs at the beginning and end of *ft*) we obtained the *ft* rate and amplitude, termed *pre* and *post*, respectively. During MVC (*pre* and *post*) in sets 1, 3, 5, and 7, we delivered a triplet PNS at the time of force’s plateau, and another triplet after 5.5s during the rest period. In sets 2, 4, 6, and 8, the stimulation during the MVC was TMS, but PNS was maintained at rest. In consecutive subjects, we reversed the order of PNS and TMS during MVC. (**b**) Example of the VA testing. Stimulation was delivered at the MVC plateau (see supporting video and figures). It is shown the force recording (up), the line of triggers (triple-PNS) (middle) and electromyographic activity. The insets on the right present an enlarged view of the twitches obtained during MVC and in the resting muscle. The Supporting Video displays the last seconds of a 30 s *ft* set, the execution of the MVC-*post* just at the end of *ft*, and the PNS (at MVC plateau and again in the resting potentiated muscle) to calculate the VA; the video was obtained with permission of the participant, in a different session from the experimental ones.

Stimulation was automatically delivered at reaching the plateau of each MVC (Fig. 1 and Supporting Fig. 3 and Video) and again after 5.5 seconds in the potentiated (resting) muscle. Stimulation in the potentiated (resting) muscle was always PNS (triplet) as well as in half of the MVC. In the other half, M1 was stimulated with a TMS single pulse (Magstim 200^2^). We used a figure-of-eight coil positioned over the *first dorsal interosseous* muscle M1 “*hot spot*”. TMS intensity was set to generate TMS silent periods (SPs) in the electromyographic activity of ≈175 ms^8^, calculated previously during MVC in the fresh muscle. TMS also served to evaluate motor evoked potential (MEP) amplitudes. Subjects 1, 3, 5 … received PNS during MVC in sets 1, 3, 5 and 7 and TMS in sets 2, 4, 6, and 8. On the other hand, subjects 2, 4, 6 … received TMS in sets 1, 3, 5 and 7 and PNS in sets 2, 4, 6, and 8; during MVC. Subjects remained at rest after executing the *post*-MVC of each set.

### Control-session

The protocol, stimulation parameters and order were the same as in the *ft session*, but subject remained at rest rather than executing *ft*. This served to evaluate the putative influence of executing MVC over time on the level of central fatigue.

A time-lag of 15 days was established between sessions, which were counterbalance in order.

### Complementary EB. Association between MVC and VA in the current set-up

Subjects executed 20 MVCs (towards flexion) with 20 s inter-repetition intervals, and the VA was tested as in the Main EB. With this, we could depict the relation between MVC and VA in our experimental setup. This EB was executed at the end of the *control session* of the main EB on the same day.

### Analysed variables

The *ft* rate and range of movement (ROM) amplitude were collected from force and goniometry sensors^8,9^ while considering the median score in the four first and last seconds of *ft* (*pre* and *post* respectively, Fig. 1a). The MVC peak force was the mean score calculated in a 10 ms time window prior to stimulation. The VA was defined as [1 − (interpolated twitch amplitude/control twitch amplitude)] · 100 ^1,2^, acquired during MVC and in the resting muscle, respectively. The TMS-SP duration was the time lag from TMS stimulation to recovery in the electromyographic activity, as determined previously^8,9^. The amplitudes of the MEP and CMAP_MVC_ (during MVC) were considered peak-to-peak (in the first of the triplet for the CMAP). We also tested the half-relaxation time in the resting potentiated twitch, which evaluates the contractile muscle properties; it increases with peripheral fatigue^10,18–20^.

### Statistical Analyses

In the Preliminary EB, the mean amplitude of the 5 rest (un-potentiated) force-twitches in flexion were compared to those obtained in abduction (paired Student t-test of absolute values; Supporting Fig. 1). For the other EB, data were processed (intra-subject normalization) as follows. For variables derived from maximal scores (*ft* rate, MVC and CMAP_MVC_ amplitude), the maximum at any testing time (considering *pre* and *post* of all sets) divided all values in the corresponding session. For *ft* ROM amplitude, the scores at *pre* and *post* in all sets were divided by the amplitude of the maximal active ROM recorded before the experiment. For the TMS-SP, MEP and half-relaxation time, the mean (across all *pre* scores) divided all scores in the corresponding session^8,9^. We used the Student t-test to test if the normalizing scores for each variable differed across sessions (Table 1).

**Table 1:** Normalizing scores for the two sessions of the main EB’s.

	*Ft* session	*Control* session	Level of significance
*ft-*rate (Hz)	7.1 (SE 0.2)	—	not applicable
Full ROM* (degrees)	50.4 (SE 2.6)	—	not applicable
MVC (kg)	4.9 (SE 0.3)	5.1 (SE 0.4)	t_10_ = 1.2 p = 0.2
SP-duration (ms)	175.6 (SE 11.1)	178.4 (SE 12.2)	t_10_ = 0.2 p = 0.8
MEP-amplitude (mV)	6.7 (SE 0.7)	6.0 (SE 0.5)	t_10_ = 0.8 p = 0.4
CMAP_MVC_ amplitude (mV)	18.7 (SE 1.2)	16.1 (SE 1.4)	t_10_ = 1.9 p = 0.1
Half Relaxation Time (ms)	51.3 (SE 1.9)	52.3 (SE 2.2)	t_10_ = 0.5 p = 0.6

^*^Maximal ROM extension from the hand resting position in the 3D fixation system.

To analyse variables in the *ft* and *control sessions*, we used repeated measures ANOVA:

- For variables acquired in all sets (MVC and half-relaxation time), factors were TASK (2 levels: *ft* and *control*), STIM (2 levels: TMS and PNS, reflecting the kind of stimulation during the MVC), SET (4 levels: sets 1–4), TIME (2 levels: *pre* and *post* task execution).
- For variables from TMS (SP duration and MEP amplitude) or PNS (VA and CMAP_MVC_ amplitude) sets, the ANOVA was TASK × SET × TIME.
- For *ft* rate and ROM amplitude, the ANOVA was STIM × SET × TIME; they were not acquired in the *control session*.

Finally, we depicted the MVC-VA and MVC- half-relaxation time relationships with regression analyses. We used scores acquired along the 20 MVC repetitions in the Complementary EB; we also included scores from the *control session* of the main EB (the *control session* did not include *ft* and was executed just prior to the Complementary EB). The independent variable was the MVC, which was pooled in 5% intervals [100-95%), [95-90%), etc., using as mark-class the mean value for each interval; the dependent variables were the VA and half-relaxation time, respectively.

In graphs, the y-axis unit is equal to the normalizing score (Table 1). Graphs represent the mean and the standard error of the mean (SEM). The normality of distributions was checked with the Kolgomorov-Smirnov test for one sample and the assumption of sphericity with the Mauchly test for ANOVA; if sphericity was violated, the degrees of freedom were corrected with Greenhouse-Geisser coefficients. A Bonferroni correction was applied for *post hoc* comparisons within the different levels of the factors. Significance was considered when p < 0.05.

### Data availability

The datasets generated during and/or analysed during the current study are available from the corresponding author on reasonable request.

## Results

Table 1 shows the normalizing scores for the *ft* and *control session*. There were no significant differences between these scores in the *ft* and *control sessions*.

### Behaviour during ft

**Figure 2:**
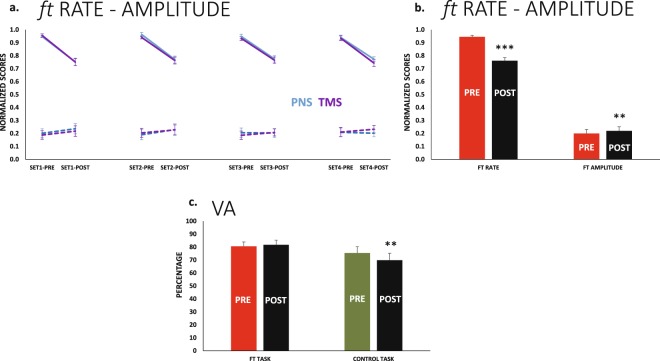
Ft rate and amplitude. Voluntary Activation (**a**) Changes from *pre* to *post* in *ft* rate (solid lines) and ROM amplitude (dashed lines). Sets corresponding to PNS during MVC are displayed in blue, and those corresponding to TMS are in purple. The profiles did not differ significantly along sets or for PNS and TMS (in these cases, we will plot results pooling sets and stimulation modes, similar to in b). (**b**) The same results in a *pre*-*post* basis. The *ft* rate decreased significantly, and the *ft* ROM amplitude presented a small but significant increase at *post*. (**c**) The VA reduced significantly at *post* only in the *control* session, this was not different in the four sets; the bars represent the four sets of each task pooled. **p < 0.01, ***p < 0.001.

The tapping rate decreased at the end of the 30 s of *ft* (F_1,10_ = 93.0 p < 0.001_TIME_) in the same way in all sets (i.e., the decreased from *pre* to *post* was not significantly different in the sets). The reduction in frequency was ≈18% (Fig. 2a,b). The ROM amplitude increased at the end of *ft*; the effect was small ≈2% but significant (F_1,10_ = 11.6 p = 0.007_TIME_) (Fig. 2a,b). Remaining main effects or interactions were not significant.

### VA and MVC changed differently for the ft and control

The VA after *ft* behaved in a significantly different way compared to *control* (F_1,10_ = 6.3 p = 0.031_TASK × TIME_). Figure 2c shows the change from *pre* to *post* in the two tasks. The VA *post ft* remained stable in all sets (p > 0.500 for main effects and interactions).

For the *control session*, the VA decreased from *pre* to *post* (F_1,10_ = 9.3 p = 0.012_TIME_), and the effect was approximately 5% in size in all sets. The set progression never had an effect on the VA; thus the drop in the VA was observed from *pre* to *post* in all sets (since the change from *pre* to *post* was not different in the 4 sets, Fig. 2c shows the scores for *pre* and *post* pooling the 4 sets).

**Figure 3:**
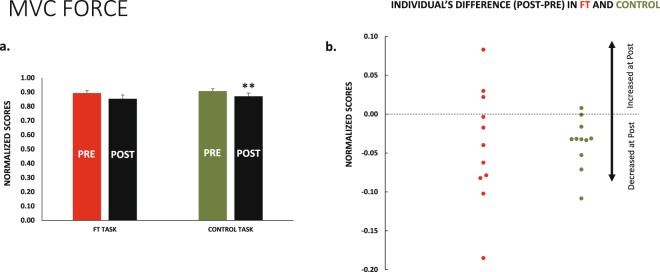
MVC force. (**a**) The muscle force during MVC was slightly reduced at *post* in the two sessions (*ft* and *control*). This was only significant in the case of the *control session*. (**b**) The small reduction in MVC force was more consistent across subjects, represented by green dots in the *control session* (red dots for *ft* session); the graphs represent all sets of each task pooled. **p < 0.01.

Likewise, the MVC change from *pre* to *post* was different in two tasks (Fig. 3a, F_3,30_ = 2.9 p = 0.051_TASK × SET × TIME_). We performed the analyses for each task (*control* and *ft*) independently, and the different responses were confirmed.

For *ft*, the force did not change from *pre* to *post* (F_1,10_ = 3.2 p = 0.105_TIME_), and the change in force from set to set showed a trend, but it was not significant (F_3,30_ = 3.7_ε__ = __0.5_ p = 0.06_SET_). Due to the relevance of this borderline effect, we performed *post hoc* analyses that indicated that MVC remained stable from set to set after *ft* (the smallest p-value for differences between sets was p = 0.2).

Conversely, for *control session*, the drop in force from *pre* to *post* was ≈4% (F_1,10_ = 14.2 p = 0.004_TIME_). It is relevant, however, than the force-drop from *pre* to *post* in the *control* was consistent across subjects; this was not the case after *ft*, though the mean reduction in force was similar in the two sessions (Fig. 3b). MVC force also decreased with set progression but only in the control session (F_3,30_ = 6.4 p = 0.002_SET_), and in this case, the mean drop from set to set was 2.8% (SEM 0.4).

### TMS-SP changed differently for the ft and control

**Figure 4:**
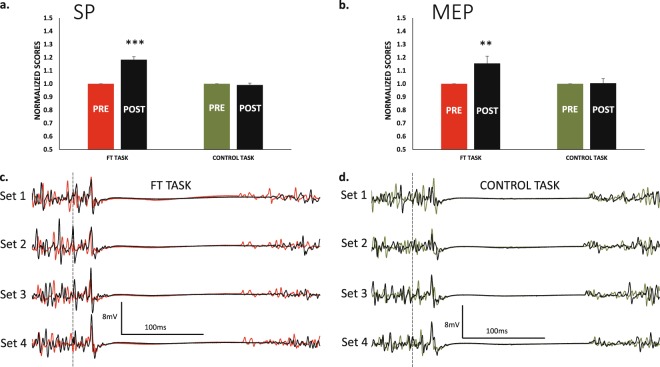
TMS SP and MEP amplitudes. For the *ft* session, the SP durations (**a**) and MEP amplitudes (**b**) increased significantly at *post*. This was not the case for the control session. In both cases, the bars represent the 4 sets of each task pooled because responses were not different in the sets. This is clearly observed at the lower section of the graph. (**c**) Recordings of the four TMS sets in a representative individual during the two sessions; *ft* at left, *control* at right. Dashed vertical lines represents the moment of delivering the TMS pulse, recordings in red for *ft* and green for *control* are *pre*, in black are *post*.

The change observed in the TMS-SP from *pre* to *post* was different for *ft* and *control* (F_1,10_ = 65.0 p < 0.001_TASK × TIME_) (Fig. 4). Follow-up analyses for the *control session* indicated that main effects or interactions were never significant (p > 0.500 for all of them), thus TMS-SP remained unchanged. For the *ft session*, the SP increased from *pre* to *post* (F_1,10_ = 61.3 p < 0.001_TIME_), and the SET effect or interactions with TIME were non-significant; thus, the increase in SP at *post-ft* was present in all sets, and its magnitude was ≈18%.

### MEP and CMAP amplitudes

CMAP and MEP amplitudes were acquired in alternative sets, and the order was counterbalanced across subjects. For MEP, we observed that amplitudes were modified differently for *ft* and *control* sessions (F_1,10_ = 5.0 p = 0.050_TASK × TIME_). Analyses by session type showed that MEP increased (≈15%) from *pre* to *post* after *ft* (F_1,10_ = 7.9 p = 0.018_TIME_); SET and SET × TIME were p > 0.05; this means that MEP increased after *ft* in all sets. MEP were unchanged for the *control* session (p > 0.05 for main effects and interactions) (Fig. 4).

**Figure 5:**
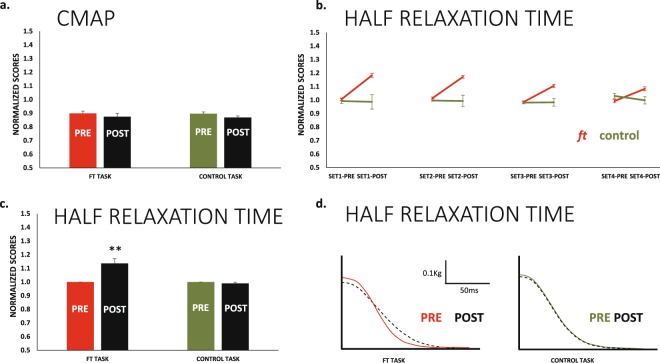
CMAP amplitudes and Half Relaxation Time. (**a**) The amplitude of the CMAP (considering the first potential of the triplet) remained stable at all testing points for both *ft* and *control* sessions. (**b**) In the *ft* session, the muscle relaxed progressively faster set after set (red lines); this effect was significant (see main text). However, in all sets, muscle relaxation was significantly slower at *post* compared to *pre* in the *ft* session. The same results represented in a *pre-post* basis in (**c**). In the case of the *control* session, these effects were absent. (**d**) Relaxation profile of the resting twitch in a same subject in the two sessions (waveforms are the average of the 8 sets at *pre* and *post*, solid and dotted lines respectively). **p < 0.01.

The CMAP amplitude never changed from *pre* to *post* or with set progressions (all main effects or interactions were p > 0.05, Fig. 5a).

### Half relaxation time. Signs of peripheral fatigue in the ft and control session

The muscle half-relaxation time in the rest potentiated twitches changed from *pre* to *post* in a different way for *ft* and *control* sessions (F_1,10_ = 14.1 p = 0.004 _TASK × TIME_). For *ft*, we observed a significant reduction in the half-relaxation time from set-to-set (F_3,30_ = 4.9_ε = __0.5_ p = 0.029_SET_), which mean change was small (≈1.5%). Conversely, within sets, the change from *pre* to *post* was much larger (≈13%), significant (F_1,10_ = 14.4 p = 0.003_TIME_) and in the opposite direction, indicating that the time of relaxation of the muscle was larger after *ft*. For the *control session*, none of these effects were present; the half-relaxation time of the muscle remained stable (Fig. 5b–d).

### Model explaining the relation MVC-VA and MVC- half-relaxation time in the present study

**Figure 6:**
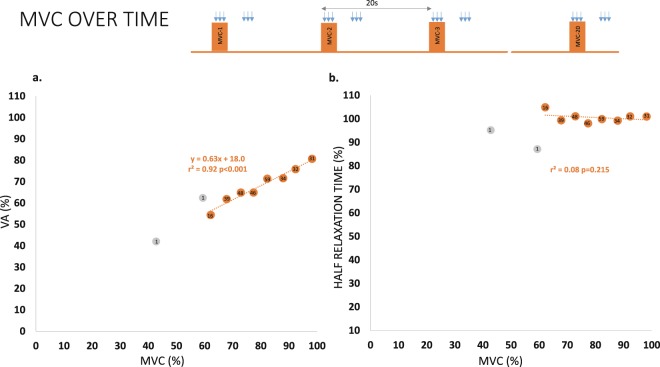
Effects of repeating MVC over time on VA and Half Relaxation Time. (**a**) The repetitions of MVC over time (executed during and after the *control session*) reduced the magnitude of force and the level of VA. The relation was linear in the range of forces tested (orange dots). (**b**) However, the drop in MVC force was not associated with the changes in half relaxation time. The score within each dot indicates the number of repetitions included for its computation (considering the 20 MVCs repeated with a 20 sec rest and the *control session* performed immediately prior to the 20 MVCs). The force ranges with a small number of events (force ranges below 60% MVC, grey dots) were not included in the computation of the regression models.

Figure 6a shows the relation of VA with MVC. It can be explained by the linear model y = 0.63·x + 18.0 (r^2^ = 0.92); this association was significant (p < 0.001). This was calculated with MVC forces in fatigued muscles displaying magnitudes above 60% of the MVC force of the unfatigued muscle. This means that the MVC-VA relation is not 1:1, but a drop of one point in MVC force reduces 0.63 points the VA in the range of forces tested. Conversely, the association between MVC and half-relaxation time was not significant (r^2^ = 0.03, p = 0.2). This is shown in Fig. 6b, where minimal changes of half-relaxation time were present with large drops of MVC.

## Discussion

This work was designed to determine if failure of central drive to the muscle has a role in the fatigue that originates during short-lasting un-resisted repetitive movements (finger tapping), executed at the maximum possible rate. Our work suggests that failure of central drive to the muscle is not a factor for the waning of maximum tapping rate along the *ft* task. Changes in MVC or VA were absent after 30 seconds of *ft*. In contrast, tapping rate decreased and MEP-amplitude, TMS-SP duration and muscle relaxation time increased after *ft*, all about 15% in magnitude.

In the *control session* without *ft*, the sequence of MVC (with the same timing as the *ft* session) decreased MVC force and VA (*pre* vs. *post*) in all sets. Likely, this means that MVC executed repeatedly with short delay (i.e., rest period between *pre* and *post* testing was 48s) generates central fatigue.

In contrast to the control session, fatiguing *ft* induced an increased inhibition of the M1 (enlarged TMS-SP at *post*). We have previously shown that this enlargement of the TMS-SP was due to its cortical component, and not to inhibition at the level of spinal cord circuits^8,9^. We also observed that TMS-MEP amplitudes increased after fatiguing *ft*, which was due, at least in part, to increased excitability of spinal cord circuits^9^. It has been previously reported that changes in the excitability of the motor cortex are not related to the modification of the central drive to the muscle during isometric contractions^21^; in agreement with that, the drop in MVC and VA from *pre* to *post* in our *control session* (including repetitions of MVC) was not accompanied by changes in MEP amplitudes nor in SP durations.

The available literature is controversial regarding VA responses to dynamic contractions. While Pasquet *et al*.^22^, showed no effects of resisted concentric or eccentric contractions on VA in the presence of MVC reduction, Yoon *et al*.^23,24^ demonstrated VA waning after tasks combining both kind of muscle activities. In our control-session MVC force and VA reduced from Pre to Post (Post 48 s after Pre). There is a general consensus that repeating MVC produces fatigue^17^, and we have also observed it in our complementary experiment including 20 MVC with 20 sec inter-repetition interval at rest. So, it appears that the time-lag between MVC at Pre and Post (i.e., 48 sec) was not the optimal one to completely avoid the (small) reductions of MVC and VA.

In agreement with Rodrigues *et al*.^6^, we observed a maintained level of MVC force after *ft*. This has not been observed in our previous works with similar methodology in which we reported MVC waning after *ft*^8,9^. Our present results indicate that the effects reported previously (with much shorter inter-set rest than in the present work) were not driven by *ft*, but likely were a consequence of repeating MVC over-time, as was observed elsewhere^25^. Altogether, our results indicate that MVC force appears to be an un-valid indicator of fatigue induced by un-resisted repetitive movements, and the same occurs with the VA. Both parameters rely on the amount of central drive to the muscle, which might be irrelevant for tasks in which the shift in movement direction is, likely, the key element. In support of this idea, Rodrigues *et al*.^6^, have suggested that fatigue during *ft* might originate in central circuits responsible for timing regulation, leading to an impaired control of the rapid changing from flexion to extension and vice-versa. Those circuits could be related to intracortical GABAergic inhibition as shown in this study. Remarkably, both timing regulation and the excitability of M1-GABAergic circuits are impaired in some diseases, such as Parkinson’s^26^. The small increase in the ROM amplitude at the end of the 30 s of *ft* observed in our study might be associated with this phenomenon (altered ability to change directions). As suggested previously, cortical and spinal excitability balance may evolve differently during fatiguing muscle contractions depending on their isometric or dynamic features^8,9^, and cortical and spinal circuits might dissociate in their role for force and time regulation, as it has been suggested previously^5,6^. This idea is favoured by the fact that silent periods induced at spinal level during MVC following fatiguing *ft* are unchanged from unfatigued conditions, whereas they enlarge if induced at cortical level. For isometric MVC fatiguing contractions the situation is different and the excitability of the spinal cord is clearly impaired, suggesting that this structure is crucial in force regulation, but less involved in changing movement directions during activities like un-resisted *ft*, for which small levels of force are required but the sequence of muscle activation is essential.

Notwithstanding, it is important to keep in mind the nature of the repetitive task tested in this work (index finger tapping) from an anatomical point of view: Direct cortico-motoneuronal connections are prominent in the control of hand muscles^27^ essential for finger tapping. However, some other rhythmic repetitive movements (like gait) are regulated by complex circuitry at the spinal cord and other sub-cortical structures^28^, which could express different fatigue profiles that the reflected in this work involving simple un-resisted repetitive finger movements of short duration. In fact, the general profile of responses obtained here differ from responses achieved with longer tasks^29,30^, especially if movements are resisted^24^. For instance, elbow flexo-extension resisted-movements executed to task-failure reduce central drive to the muscles^24^.

In our study, the absence of reduction of the voluntary drive in the presence of *ft* rate waning cannot be attributed to the experimental set-up. The support for this statement is twofold. First, VA levels in the un-fatigued conditions were the expected values with reference to the literature reports, either considering empirical data^31^ or modelling^32^. Second, when fatigue developed by repetitions of MVC over time (without *ft*), we showed a strong lineal relationship between MVC reduction and central drive waning. A previous work exploring this relation in elbow flexors had shown that it followed a logarithmic function^33^; however, that work and ours differed in the muscles and force ranges explored. In our work, the linear relation of VA/MVC was 0.6/1. This is likely the reason why the *control session* showed significant drops in MVC and VA from *pre* to *post* but not from set-to-set: the magnitude of MVC drop was small set-after-set (approximately 50% the observed in *pre*-*post* comparison).

In contrast to the abovementioned small (but significant) effects on VA and MVC in absence of *ft*, in the *ft-session* the responses of other fatigue-related parameters are clear-cut. The maximal *ft* rate dropped by ≈18% in 30s. In parallel, the excitability of inhibitory cortical interneurons increased about ≈18% and corticospinal excitability raised ≈15%. This fits well with previous results which showed that the increase in SP after fatiguing *ft* is due to its cortical but not to its spinal component^8,9,34–36^. Along with the increased excitability of M1-GABAergic circuits, the increased cortico-spinal excitability (MEP amplitudes) might serve as a compensatory mechanism that could take place at the level of spinal cord circuits^9^. This possibility is in line with results reported by others, where 10 sec of maximal rate *ft* increased corticospinal excitability and also the excitability of cortico-cortical inhibitory interneurons^5^.

A particularly relevant result of our study is the slowing of muscle relaxation (SMR) at the end of *ft*, reflected as an increased in the half-relaxation time. This is a marker of peripheral fatigue, which is known to develop faster than central fatigue during dynamic contractions^12,37^. Unlike what has been shown for isometric activities, where SMR is seen as a mechanism contributing to lessen force loss when motor unit firing rate decreases^10,18,19^, here, SMR seems to be a contributing factor to the reduced tapping rate in the repetitive movements^10^. In our protocol, SMR was tested after an MVC, but we trust it was induced by *ft* because in the *control session* (without *ft*), the velocity of muscle relaxation did not slow^38^. Perhaps, for this reason, the reduction in MVC was much more consistent in our *control session*. Muscle relaxation after *ft* was slower in all sets, but muscle relaxed interestingly faster with set progression (either at *pre* and *post*, though *post* remained always slower). This effect (only present in the *ft session*) is likely related to temperature increases induced by the repetitive nature of *ft*^39^. Currently, there is a debate regarding the validity of the twitch-interpolation technique to test central fatigue in the presence of SMR^32^. Some reports indicate that SMR contributes to overestimating the level of VA^40^, while others exclude this possibility^41^. In our case, the SMR increased after *ft*, but we cannot establish a causal link for VA behaviour. This is because a contributing factor for central drive to the muscle (like corticospinal excitability, i.e., MEP, which is due to spinal excitability at least in part^9^) was also increased *post-ft*. Regardless the mechanism, our results suggests that the estimation of the central drive to the muscle with twitch-interpolation technique appears to be not valid to detect the early expressions of neural adaptations induced by fatiguing un-resisted repetitive movements, such as finger tapping.

Fatigue has gained importance as a clinical sign in many pathologies^42^. Currently, the objective evaluation of fatigue in many clinical entities is mostly relying on tests involving force/isometric tasks, although many activities of daily living involve muscle contractions other than isometric ones. Our data suggest that such an approach might be insufficient for detecting central expressions of fatigue in tasks other than those that are isometric or dynamic activities requiring high levels of force. Whether the increased inhibition at a cortical level reflects a central adaptation to avoid the overloading of a poorly functional muscle or are independent phenomena must be clarified in the future. This possibility has been confirmed^43^ and refuted^44^ for intermittent and sustained MVC, respectively.

### Study Limitations

Two issues are worth considering here. Firstly, we have evaluated the expressions of fatigue induced by *ft* by acquiring motor potentials from the superficial head of the FDI muscle and by stimulating the ulnar nerve, while the thumb was secured in abduction. Our experiments presented in the supplementary information shows the suitability of this set-up for our purposes. However, several muscles contribute to a single joint task, therefore the global expression of fatigue associated to *ft* at the maximal rate might be different to the observed specifically in the FDI muscle.

Secondly, we have included a *control-session* for testing fatigue parameters (with the same timing of events as the *ft-session*), where small but significant changes in fatigue measures occurred over a 48 s period of inactivity, likely product of the repeated execution of MVC. Nevertheless, we believe that this condition is a true “*control*” in the context of our study, since allows the comparisons of after-effects specifically induced by *ft*. However, in future experiments, it should be carefully considered to include larger rest periods to test VA over time, even in the cases of maximal efforts of short duration in small muscles.

## Conclusion

Fatigue induced by short-lasting repetitive un-resisted finger movements may have peripheral and central expressions, but within its neural components, the reduction of the central drive to the muscle appears to be excluded.

## Electronic supplementary material

Supporting Video

Supporting Figures

Supporting Sketch Files
