# Publisher Correction: Effects of a Finger Tapping Fatiguing Task on M1-Intracortical Inhibition and Central Drive to the Muscle

**DOI:** 10.1038/s41598-018-31223-w

**Published:** 2018-08-29

**Authors:** Antonio Madrid, Elena Madinabeitia-Mancebo, Javier Cudeiro, Pablo Arias

**Affiliations:** 1 Universidade da Coruña, NEUROcom (Neuroscience and Motor Control Group) and Biomedical Institute of A Coruña (INIBIC), Department of Biomedical Sciences, Medicine and Physiotherapy-INEF Galicia, A Coruña, Spain; 2 Centro de Estimulación Cerebral de Galicia, A Coruña, Spain

Correction to: *Scientific Reports* 10.1038/s41598-018-27691-9, published online 19 June 2018

A supplementary zip file containing Supporting Sketch Files was omitted from the original version of this Article. This has been corrected in the HTML version of the Article; the PDF version was correct at time of publication.

