# Supplementary material for: Effects of a Finger Tapping Fatiguing Task on M1-Intracortical Inhibition and Central Drive to the Muscle
Source: Sci Rep. 2018 Jun 19;8:9326. doi: 10.1038/s41598-018-27691-9 (PMC6008331; doi:10.1038/s41598-018-27691-9)
Supplement: Supplementary file 2 — Supporting Figures [file 41598_2018_27691_MOESM2_ESM.pdf]

SUPPORTING FIGURES

EFFECTS OF A FINGER TAPPING FATIGUING TASK ON M1-INTRACORTICAL INHIBITION AND CENTRAL DRIVE TO THE MUSCLE

Antonio Madrid[1], Elena Madinabeitia-Mancebo[1], Javier Cudeiro[1-2] and Pablo Arias[1]

[1]Universidade da Coruña, NEUROcom (Neuroscience and Motor Control Group) and Biomedical Institute of A Coruña (INIBIC). Department of Biomedical Sciences, Medicine and Physiotherapy-INEF Galicia, A Coruña, Spain

[2]Centro de Estimulación Cerebral de Galicia. A Coruña, Spain

Corresponding Author: Pablo Arias (pabloarias.neurocom@udc.es)

Supporting Figures

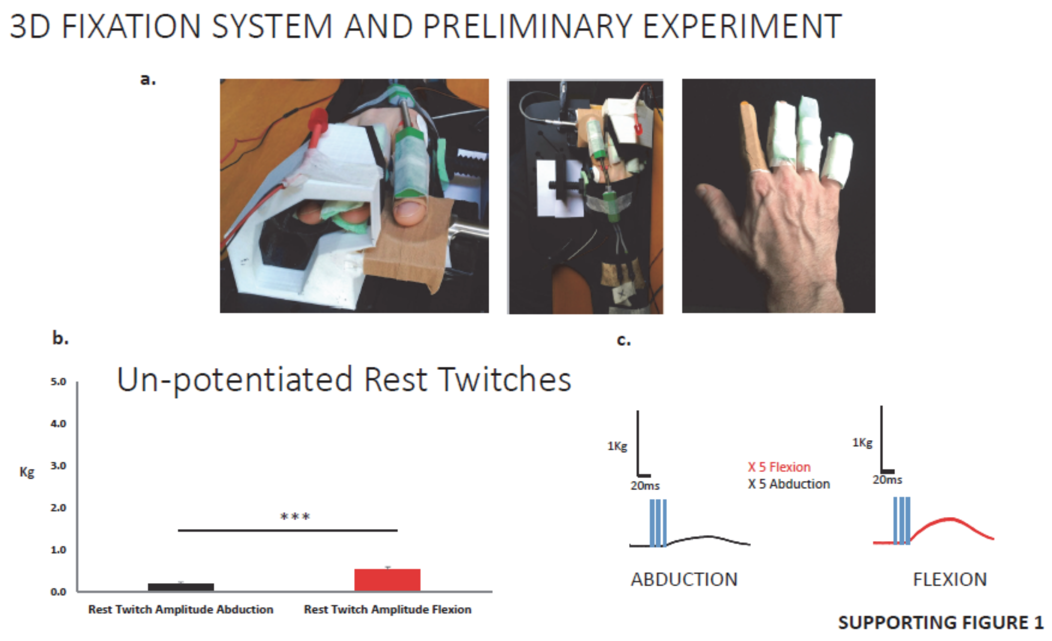

Supporting Fig 1. *3D-fixation system setup and force induced by percutaneous ulnar nerve stimulation in the un-potentiated resting muscle*

a) The 3D-fixation system secured the subjects' dominant hands. The forearm was resting on the system with the palm of the hand on a fixation anatomical support (with the wrist slightly extended, ≈25°). The thumb was abducted and its metacarpal bone secured and immobilized with the help of the system screw. The 3rd to 5th fingers were introduced and immobilized in another segment of the system, and straps were used to secure the dorsal side of the hand as well as the wrist and forearm. A dynamometer (P200 Biometrics Ltd) was adapted and secured to the system, placed flat underneath the distal phalange of the index finger in the main experiments. The index finger was free to move around the metacarpophalangeal joint (at rest, in touch with the dynamometer, it was flexed ≈45°), but its interphalangeal joints were immobilized with a finger splint. In preliminary sessions, the dynamometer was also placed and secured in a vertical position (see Supporting Sketch Drawings). This way the dynamometer was in contact with the lateral side of the distal phalange of the index finger. In the preliminary session, five supramaximal PNS triplets were delivered with the dynamometer in each position. In the preliminary session, it was clear that the stimulation produced a prominent force towards flexion (significantly greater than abduction, t_{10}=9.9 $p<0.001$). This is shown for all subjects **(b)** and for a representative participant **(c)** (the 5 traces are overdrawn; the blue vertical lines represent PNS). This constituted a basic pre-requisite to use the setup for testing the VA in our main experiments.

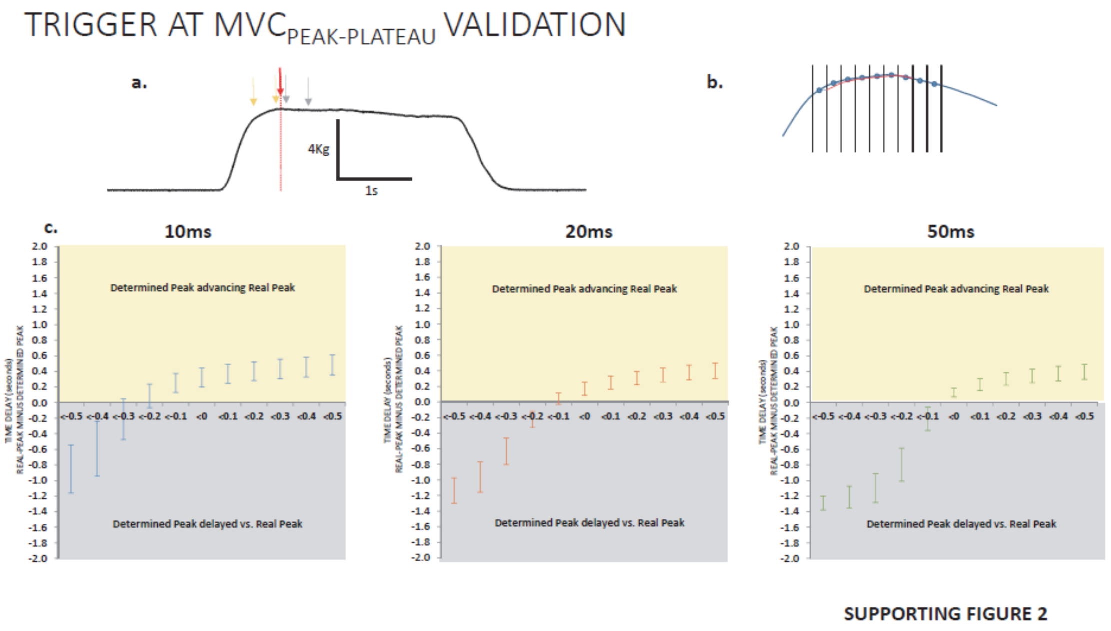

Supporting Fig 2. *Preliminary Validation of algorithms to trigger stimulation at MVC peak-plateau*

Subjects executed 15 MVCs of 3 seconds without stimulation with one minute rest. Across subjects, the sequence of MVC produced a mean reduction in force of approximately 30% to the 71.3% (SE 2.3) of the maximal MVC force. **a.)** MVC peaks were obtained *off-line* and saved as *gold-standard* (red arrow in the example) to compare MVC_{peak} determinations of different algorithms (the yellow arrows are examples of determinations advancing the *gold standard*; grey arrows delayed from the *gold standard*). Algorithms determined peaks *on-line* when the slope of the ongoing force signal was below *m* with bins (*b*) of 10, 20, and 50 ms to calculate it. **b)** For a bin of 10 ms (black vertical thin lines), the algorithm calculated the mean value of the force for that bin and stored it (b_x), doing the same operation 10 ms after (b_{x+10ms}) (blue dots). Next, a line intercepting the values b_x and b_{x+10ms} at the end of the corresponding bin was determined, and its slope calculated (red solid lines); when the slope was below *m*, the algorithm considered that the peak of force was reached. At this point, stimulation would be delivered. A threshold was set to apply the algorithm only above certain levels of force; this was done to avoid the computation of force values corresponding to *ft.* **c)** Different combinations of *m*=[<-0.5,<0.5] with 0.1 increments (abscise axis), and *b*=10, 20 and 50 ms were checked (one plot for each *b*, from left to right). We calculated the variable "time of gold-standard MVC_{peak} *minus* time of estimated MVC_{peak}" considering all MVC and subjects, and the 95% confidence interval (CI) of the mean of the differences was computed. To be used in main EB, we selected the combination of *m* and *b* yielding the narrowest 95% CI containing 0; this means no significant difference between real and determined peaks. These were *m*=[<-0.1] *b*=[20 ms].

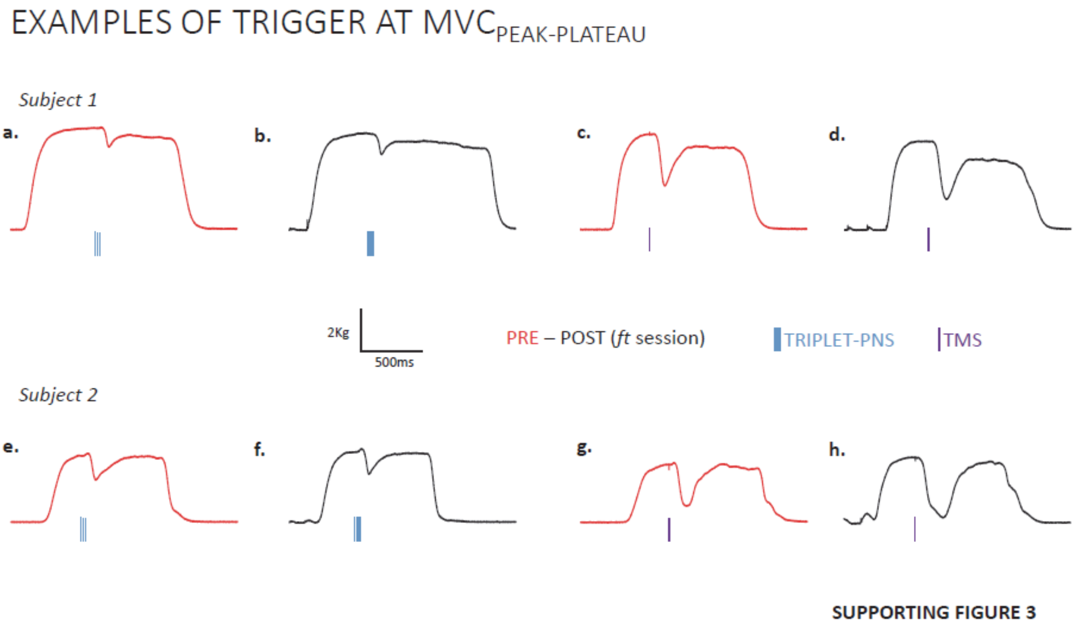

Supporting Fig 3. *Examples of stimulation at MVC peak-plateau in the main experiments*

Examples of force recordings during MVC (*pre* in red and *post* in black) in two different subjects (upper and lower traces). For each subject, the *pre* and *post* force recordings are shown for the same set including PNS (**a-b** and **e-f**) and TMS (**c-d** and **g-h**). The vertical lines represent the timing of the triplet-PNS, and single pulse TMS. It is observable the inhibition in force development with stimulation (much larger for TMS). Both subjects are representative of different levels of force and VA. In all cases, the delivering of stimulation at the plateau of force and the subsequent force inhibition produced by stimulation are clear.
